# The Effects upon the Royal Infirmary, Bradford

**Published:** 1912-01-13

**Authors:** J. J. Barron

**Affiliations:** the Secretary-Superintendent.


### A SYMPOSIUM OF HOSPITAL WORKERS.

**[We invite correspondence from our readers and all interested in the probable effects of the National Insurance Act on the Voluntary Hospital system. All letters should be accompanied by the name and address of the writer, but not necessarily for publication.]**

### THE EFFECTS UPON THE ROYAL INFIRMARY, BRADFORD.

By Mr. J. J. Barron, the Secretary-Superintendent.

Sir,—I beg to state my views on the probable effects of the National Insurance Bill on Voluntary Hospitals, and of the awkward position in which the Voluntary Hospitals, their staffs, and those requiring their help will be undeservedly placed.

### Disastrous Effects upon the Hospital Finances.

In the first place, I fear that the finances of these institutions will be disastrously affected by the passing of this Bill, as the two principal sources of revenue—subscriptions from employers and workpeople—will, almost without doubt, show a considerable falling off when the Act comes into operation. At the Bradford Royal Infirmary the total ordinary income last year was £9,834, and, in addition, we received £2,100 as the third instalment of a Five Years' Guarantee Fund which will expire in October 1912. The total expenditure was £12,981, and £1,350 had to be taken from the deposit account to meet the deficit on the ordinary account.

In view of the Special Maintenance Fund expiring next year, a special effort has been made during 1911 to obtain new and increased annual subscriptions, and for some months this effort was rewarded with very gratifying success. Recently, however, we have found it increasingly hard to get either new or increased contributions, and in many cases have had great difficulty in obtaining renewals of annual subscriptions. The prospect of the Bill becoming law caused some former supporters to cease contributing and others to withhold their subscriptions pending the passing of the National Insurance Act, while others have given notice that on the Act becoming law they will remove their names from our list of contributors.

Altogether it is impossible at present to estimate what the loss of income will be, though the amount will be considerable, as the Act will press very heavily on an industrial community like that of Bradford. I am informed that some of the large firms in this district will have to pay from £1,000 to £2,500 per annum as employers' contributions under the Bill, and I find it difficult to believe that Voluntary Hospitals will, in the future, receive from employers the same amount of support as they have done in the past. The workpeople, after paying threepence or fourpence a week for medical benefit, will not be inclined to continue the weekly or monthly system of collections for the Voluntary Hospitals, though they will ultimately see that it will be in their own interests to do so.

### The Building Fund at a Standstill.

The Building Fund for our proposed new Royal Infirmary has been adversely affected by the Insurance legislation and the fund is practically at a standstill, for, although the supporters of the present institution may continue their subscriptions in whole or in part, they do not feel called upon to promise donations for the building of a new infirmary under the present uncertain conditions. The feeling is being freely expressed here that the Act will inevitably result in the Voluntary Hospitals being taken over by either the Municipalities or the State, as funds will not be forthcoming otherwise for their upkeep, and it is recognised that the medical benefit provided under the Act is bound to prove totally inadequate for the needs of those insured, thus throwing more work on, which cannot be done except by agreement with, the medical charities.

It is not easy to estimate the amount of present in-patient accommodation which the Voluntary Hospitals will be unable to maintain, as so much must depend on the support received and the amount of interest accruing from investments for the upkeep of endowed wards and beds. In the Bradford Royal Infirmary the interest on the amounts given for this purpose produces about £1,400, and the other investments return about £900 annually, making a total dependable income of rather more than one-sixth of the amount required for the annual maintenance of the institution.

### The Position of the Insured and the Additional Hardships before Them.

Consideration of the eligibility, or otherwise, of insured persons to free hospital benefit is not made much easier by Mr. Lloyd George's statement that such persons "will have no right to come to the hospitals." The provision in the Bill for the treatment of serious medical or surgical cases is ludicrously inadequate, and such cases will as much as before require hospital treatment. To refuse to admit workpeople in urgent need of the medical or surgical assistance which they cannot obtain in their own homes from the Medical Officers appointed under the Act will entail much undeserved suffering upon them, and yet I cannot see how the Voluntary Hospitals can continue to accept those cases if the funds are not forthcoming to provide the requisite treatment. Should those cases come with the usual letter of recommendation from a subscriber, the hospitals would be bound to do the best they could for them, but if, as I fear, the number of subscribers, and therefore of recommendations, is appreciably diminished because of the Act, the lot of the more seriously ill workers will be most unfortunate. At present, with 210 general hospital beds in Bradford, we have a weekly list of from thirty-five to fifty cases waiting for admission, and the probability of the number of beds being reduced through lack of funds for maintenance must inevitably tend to still further increase this list, with the result that only the most serious cases can be admitted, and many of these only after a long period of waiting. The possibility of the increased suffering and hardship caused to those unfortunate people cannot be viewed with equanimity by any humane person, and least of all by those who know the necessity for early treatment in serious medical and surgical conditions.

It is to be hoped that the workpeople (for whose benefit the Voluntary Hospitals exist) will not be placed in the unhappy position I have described, and that the hospitals, with their great humanitarian, scientific, and teaching traditions will be allowed to proceed with their work unhampered by the fear of lack of funds to carry it on; while the members of the Boards of Management who have so freely devoted their time, money, and experience for the benefit of their more unfortunate fellow-citizens, may continue that service which has done so much to make these institutions what they are to-day—the best of their kind.—I am, yours faithfully,

J. J. Barron,
Secretary-Superintendent.

